# The potential value of exosomes as adjuvants for novel biologic local anesthetics

**DOI:** 10.3389/fphar.2023.1112743

**Published:** 2023-01-26

**Authors:** Yunmeng Zhang, Shangzhi Feng, Xin Cheng, Kecheng Lou, Xin Liu, Ming Zhuo, Li Chen, Junming Ye

**Affiliations:** ^1^ The First Clinical College, Gannan Medical University, Ganzhou, Jiangxi, China; ^2^ Department of Anesthesiology, The First Affiliated Hospital of Gannan Medical University, Ganzhou, Jiangxi, China; ^3^ Department of Urology, The First Affiliated Hospital of Gannan Medical University, Ganzhou, Jiangxi, China

**Keywords:** local anesthetic adjuvants, adverse reactions, organ remodeling, engineered extracellular vesicles, regional blockade

## Abstract

The side effects of anesthetic drugs are a key preoperative concern for anesthesiologists. Anesthetic drugs used for general anesthesia and regional blocks are associated with a potential risk of systemic toxicity. This prompted the use of anesthetic adjuvants to ameliorate these side effects and improve clinical outcomes. However, the adverse effects of anesthetic adjuvants, such as neurotoxicity and gastrointestinal reactions, have raised concerns about their clinical use. Therefore, the development of relatively safe anesthetic adjuvants with fewer side effects is an important area for future anesthetic drug research. Exosomes, which contain multiple vesicles with genetic information, can be released by living cells with regenerative and specific effects. Exosomes released by specific cell types have been found to have similar effects as many local anesthetic adjuvants. Due to their biological activity, carrier efficacy, and ability to repair damaged tissues, exosomes may have a better efficacy and safety profile than the currently used anesthetic adjuvants. In this article, we summarize the contemporary literature about local anesthetic adjuvants and highlight their potential side effects, while discussing the potential of exosomes as novel local anesthetic adjuvant drugs.

## 1 Introduction

Anesthetic drugs represent one of the greatest medical discoveries of all time (Eckenhoff et al., 2008). The use of anesthesia avoids the occurrence of severe intraoperative and postoperative physiological reactions, including severe fluctuations in blood pressure, respiratory irritation, and local muscle spasms, which are triggered by the surgical trauma and the associated stress response. Second, intraoperative unconsciousness and amnesia help minimize the occurrence of postoperative psychological disorders (Rabbitts and Kain, 2019). General anesthesia and regional anesthesia are the two main types of anesthesia used in surgical practice. An estimated 40 million patients per year are subjected to anesthesia in the United States alone. Local anesthesia is only used for small or local anesthesia-only procedures (Pincus, 2019). Since patients can more efficiently transition to continuous care and can maintain their breathing status with faster postoperative recovery, this avoids invasive injuries and the occurrence of postoperative delirium and cognitive impairment due to anesthetic manipulations such as tracheal intubation (Xu et al., 2018). As a result, the indications for local anesthesia have been gradually expanded. However, adverse reactions to local anesthetic drugs have also become a concern such as pulmonary, renal, neurological, and cardiovascular complications. Besides, malignant hyperthermia and allergic reactions are relatively uncommon but have serious consequences. The development of local anesthetic adjuvants to avoid or mitigate these complications may be one of the effective safeguards for the clinical use of anesthetic drugs. Currently, some commonly used drugs such as dexamethasone, dexmedetomidine, and neostigmine are increasingly being used as anesthetic adjuvant drugs; these drugs have effectively reduced the burden of care and improved patient satisfaction as well as the safety and efficiency of anesthetic drugs.

Yet, there are many unaddressed issues related to these anesthetic adjuvant drugs, particularly with respect to their uncertain efficacy and their own side effects. For example, the immunosuppressive effect of dexamethasone may increase the risk of the spread of infection. Dexmedetomidine has been shown to exacerbate nerve damage in patients with diabetic neuropathy (Lirk and Brummett, 2019). Neostigmine, an anticholinesterase drug, has been shown to exacerbate clinical symptoms in patients with bradycardia (Lirk and Brummett, 2019). This suggests the need for further research on the development of novel local anesthetic adjuvants. However, there has been little progress in this field and the classes of anesthesia-assisting drugs used clinically have not changed in the last decade. Most investigators believe (and hope) that anesthetic adjuvants can mitigate the side effects of anesthetic drugs by interacting with specific proteins through unique binding sites. However, the existing adjuvants represent more of a diversification of drugs, which can themselves cause many serious side effects. In addition, the anesthetic adjuvants developed in response to specific clinical symptoms do not significantly ameliorate the damage to the parenchymal organs caused by anesthetic drugs (Prabhakar et al., 2019). Chemical-physical properties of adjuvants can cause bio-incompatibility, skin and vascular irritation, and low drug utilization (Cyske et al., 2021). All these suggest that the research on new anesthetic adjuvants should focus on new molecular targets, bioactive agents, and protection against parenchymal organ damage. Exosomes, a representative class of new bioactive substances, has evoked the interest of researchers. Exosomes released from specific cells have shown some unique effects in repairing damaged tissues and organs. For example, exosomes from mesenchymal stem cells can improve neural cell damage and thus reduce the neurotoxicity of anesthetic drugs (Gu and Zhu, 2021); exosomes of cerebrospinal fluid origin can promote the proliferation of neuronal cells *in vitro* and thus participate in the neuronal repair process (Kong et al., 2018); and exosomes released from cardiomyocytes after exercise have a powerful cardioprotective effect (Hou et al., 2019). These results suggest the great potential of exosomes as anesthetic adjuvant drugs. Exosomes have also shown great potential in alleviating postoperative complications, such as reducing postoperative pain (Cata et al., 2022), improving sleep, cognitive impairment (Hsu et al., 2020; Gu and Zhu, 2021), and reducing the frequency of arrhythmias (Poon et al., 2017; Ayala-Mar et al., 2021). Therefore, exploring specific sources of exosomes as anesthetic adjuvants may be a new avenue for future development of biological anesthetic adjuvants.

## 2 Local anesthetic adjuvant and complications

Since local anesthesia adjuvants have brought benefits to local anesthesia surgery and general anesthesia surgery, physicians have become concerned about patient satisfaction, speed of recovery, operator convenience, and patient safety (Prabhakar et al., 2019). This has led to the development of new local anesthetic adjuvants that are more inclined to address the clinical adverse reactions of current anesthetic drugs, such as reducing postoperative pain, shortening awakening time, accelerating recovery of gastrointestinal function, and increasing the speed of incisional healing. Studies have shown that the currently used local anesthetic adjuvants can indeed help accelerate the onset of action of anesthetic drugs, prolong intraoperative and postoperative analgesia, improve analgesia quality, and reduce potential adverse drug reactions (Prabhakar et al., 2019). The most common of these are non-opioid local anesthetic adjuvants such as dexamethasone, midazolam, alpha-2 agonists (colistin and dexmedetomidine), NMDA antagonists (including ketamine and magnesium), neostigmine, sodium bicarbonate, and epinephrine drugs (Prabhakar et al., 2019). In addition, several other local anesthetic adjuvants are being developed and used (Wiles and Nathanson, 2010), such as adenosine, glucan, opioids, and non-steroidal anti-inflammatory drugs (NSAIDs) (Table 1). However, local anesthetic adjuvants also pose many hazards, such as neurotoxicity, gastrointestinal reactions, and cardiovascular risks (Sun et al., 2017; Zhang et al., 2017; Yu et al., 2019). Below, we describe the use of existing local anesthetic adjuvants and focus on their potential risks in order to construct a suitable strategy for the development of novel local anesthetic adjuvants.

**TABLE 1:** Effectiveness and potential side effects of anesthetic adjuvants.

Drugs	Type	Effect as anesthesia adjuvant drugs	Side effect	References
Corticosteroids	Dexamethasone	Prolongs nerve blockade time, Reduces pain intensity, Reduces sleep disturbances	Immunosuppressed, Increased risk of infection, Affects wound healing	Leurcharusmee et al. (2016); Kheirabadi et al. (2020)
Benzodiazepines	Midazolam	Prolongs analgesic time and reduces adverse reactions; Sedation and forgetfulness	Respiratory depression, Neurotoxicity	Shibuta et al. (2018); Amin et al. (2020); Xu et al. (2021)
Alpha2 agonist	Clonidine, Dexmedetomidine	Prolong sensory and motor blockade	Hypotension, Bradycardia, neurotoxicity	Mostafa et al. (2020); Perez-Zoghbi et al. (2020); Chen et al. (2022)
NMDA antagonist	Ketamine, Magnesium	Prolong the duration of anesthesia and reduce postoperative analgesic drug consumption, Reduce chills	Hypotension, Lethargy, Muscle weakness, Respiratory depression, Arrhythmias, Hallucinations, Memory loss, Nausea, Vomiting	Gozdemir et al. (2010); Na et al. (2010); Coull et al. (2011)
Neuromuscular-blocking drugs	Neostigmine	Relieves postoperative pain	Hypotension, Bradycardia, Nausea, Vomiting	Mahajan et al. (2004); Kumari Vasantha and Madhusudhana (2018)
Sodium bicarbonate	—	Shortens the onset of action of analgesia, Relieves injection pain	Electrolyte abnormalities	Christoph et al. (1988); Saatchi et al. (2015); Phero et al. (2017)
Epinephrine	—	Reduces postoperative pain intensity, Delays the toxic reaction caused by local anesthetic entering the blood vessel	Ischemia of organ tissues	Corvetto et al. (2012); Aljahany et al. (2020); Sallum et al. (2022)
Non-steroidal anti-inflammatory drugs	Acetylsalicylic acid, Etorolac	Enhance the analgesic effect, reduce postoperative opioid consumption	Organ damage: gastric mucosal damage; cerebral hemorrhage; kidney damage; liver toxicity	Corpataux et al. (1997); Tsujimoto et al. (2018); Wagner-Kovacec et al. (2018)
Opioids	Buprenorphine, Ramadol	Reduce pain intensity	Pruritus, Urinary retention, Nausea, Delayed respiratory depression	Mahendru et al. (2013); Nayagam et al. (2014)
Adenosine	—	Anti-inflammatory and analgesic	Headache, Arrhythmia	Al-Mallah et al. (2011); Ammar et al. (2018); Haanes et al. (2018)
Glucan	Dextran	Prolonges analgesic time, Increases local anesthetic effect	Drug allergic reaction	Navaratnarajah and Davenport (1985); Zinderman et al. (2006); Tsuchiya et al. (2018)

The advantage of existing local anesthetic adjuvants is that they prolong the duration of anesthesia and reduce pain intensity. Dexamethasone is a long-acting synthetic corticosteroid that reduces the inflammatory response to tissue injury (Prabhakar et al., 2019). A meta-analysis compared the analgesic effects of dexamethasone by both perineural and intravenous routes of administration. Application of dexamethasone was shown to reduce pain scores and pain intensity in clinical studies (Biradar et al., 2013). Intrathecal application of midazolam facilitates the action of local anesthetics (Salimi et al., 2014). Interestingly, a new composite material consisting of poly electrospun fibers of ropivacaine and colistin-containing hydrogel was used in a rat sciatic nerve block model. This anesthetic composite was found to accelerate the onset of action and allow sensory and motor blockade for more than 32 h, thus significantly prolonging the duration of analgesia compared to the single application of ropivacaine (Chen et al., 2022). Colistin also has a sedative effect, but unlike midazolam, it achieves sedation primarily by decreasing sympathetic nervous system activity and level of consciousness (Fernandes et al., 2018). As an adjuvant drug for intrathecal or epidural anesthesia, magnesium may prolong the duration of anesthesia and reduce intraoperative anesthetic requirements and postoperative analgesic drug consumption (Kutlesic et al., 2017). The addition of neostigmine in the spinal cord leads to anti-injury perception and attenuation of pain. Intrathecal injection of neostigmine in rats was found to attenuate acute postoperative pain in a dose-dependent manner (Huang et al., 2022). Guo et al. found that the addition of sodium bicarbonate and epinephrine to lidocaine in inferior alveolar nerve blocks reduced injection pain and shortened the duration of analgesic episodes (Guo et al., 2018). Epinephrine as an adjunct to upper extremity peripheral nerve blocks in pediatric patients was shown to prolong the duration of sensory and motor blocks and reduce the need for analgesics (Mikjunovikj-Derebanova et al., 2021). In a study, the addition of ketorolac (an NSAID) to levobupivacaine improved analgesic efficacy and reduced postoperative opioid consumption at 24 h and 48 h compared with levobupivacaine alone in cesarean wound infusion (Wagner-Kovacec et al., 2018). In abdominal surgery, intrathecal opioids were found to reduce postoperative pain scores at rest and during exercise and shorten the length of hospital stay (Bacchi et al., 2012). In a systematic review exploring the effectiveness of adenosine for postoperative analgesia, adenosine was found to reduce postoperative pain for 24 h compared to remifentanil (Jin and Mi, 2017).

However, the adverse effects of local anesthetic adjuvants such as respiratory inhibition and neurotoxicity has led to limitations in their use. The use of dexamethasone is associated with certain adverse effects, including immunosuppression, increased risk of infection, and impaired wound healing (Percival et al., 2010). The neurotoxicity of midazolam has been widely noted (Shibuta et al., 2018). Some *in vitro* studies have shown the potential neurotoxicity of midazolam for perineural blockade. An experiment investigating the neural blockade and neural mechanisms induced by midazolam in a rat model found more pronounced neurotoxicity of midazolam, which may be related to the midazolam-induced release of calcium ions stored inside sensory neurons (Yilmaz et al., 2014). Studies evaluating the safety and efficacy of dexmedetomidine in nerve blocks have found an increased risk of hypotension and bradycardia with dexmedetomidine (Schnabel et al., 2018). Ketamine use is associated with notable adverse effects, including hallucinations, memory loss, nausea, vomiting, and cardiovascular system stimulation (Niesters et al., 2014). Neostigmine has some potential value in relieving pain and reducing side effects associated with opioids, but its clinical use is limited by its gastrointestinal side effects. Its potential adverse effects include sweating, bradycardia, irritability, and severe nausea and vomiting (Swain et al., 2017). In one study, intrathecal bupivacaine combined with neostigmine was found to cause severe nausea and vomiting (Kumari Vasantha and Madhusudhana, 2018). Sodium bicarbonate can alter intracellular and extracellular pH and may cause electrolyte disturbances. The use of epinephrine can affect circulatory stability, which may trigger a multi-organ ischemic response (Ilicki, 2015; Van Demark et al., 2021). The side effects of NSAIDs are also well known, including gastrointestinal toxicity, cardiovascular risk, renal injury, and hepatotoxicity (Bindu et al., 2020). The use of opioids is associated with certain adverse effects, such as pruritus, urinary retention, and nausea (Sun et al., 2017). Adenosine also has some side effects including panic, headache, and transient atrioventricular block, but substantial studies are lacking to confirm this (Andrikopoulou et al., 2019; Thuraiaiyah et al., 2022).

In summary, several drugs have been used clinically as adjuvants, and their addition has been shown to contribute to improved patient safety, patient satisfaction, and enhanced clinical outcomes. However, the advantages of their clinical use are not significant compared to their potential risks. Therefore, further research is required to maximize the value of local anesthetic adjuvants in clinical use (Edinoff et al., 2021). Biologically active drugs have been successfully used in many fields in recent years and have higher efficiency, bioaffinity, and targeting properties compared to existing chemosynthetic drugs. Therefore, biologically active drugs may represent an important direction for the development of new local anesthetic adjuvants.

## 3 The potential value of exosomes as a local anesthetic adjuvant

**FIGURE 1:**
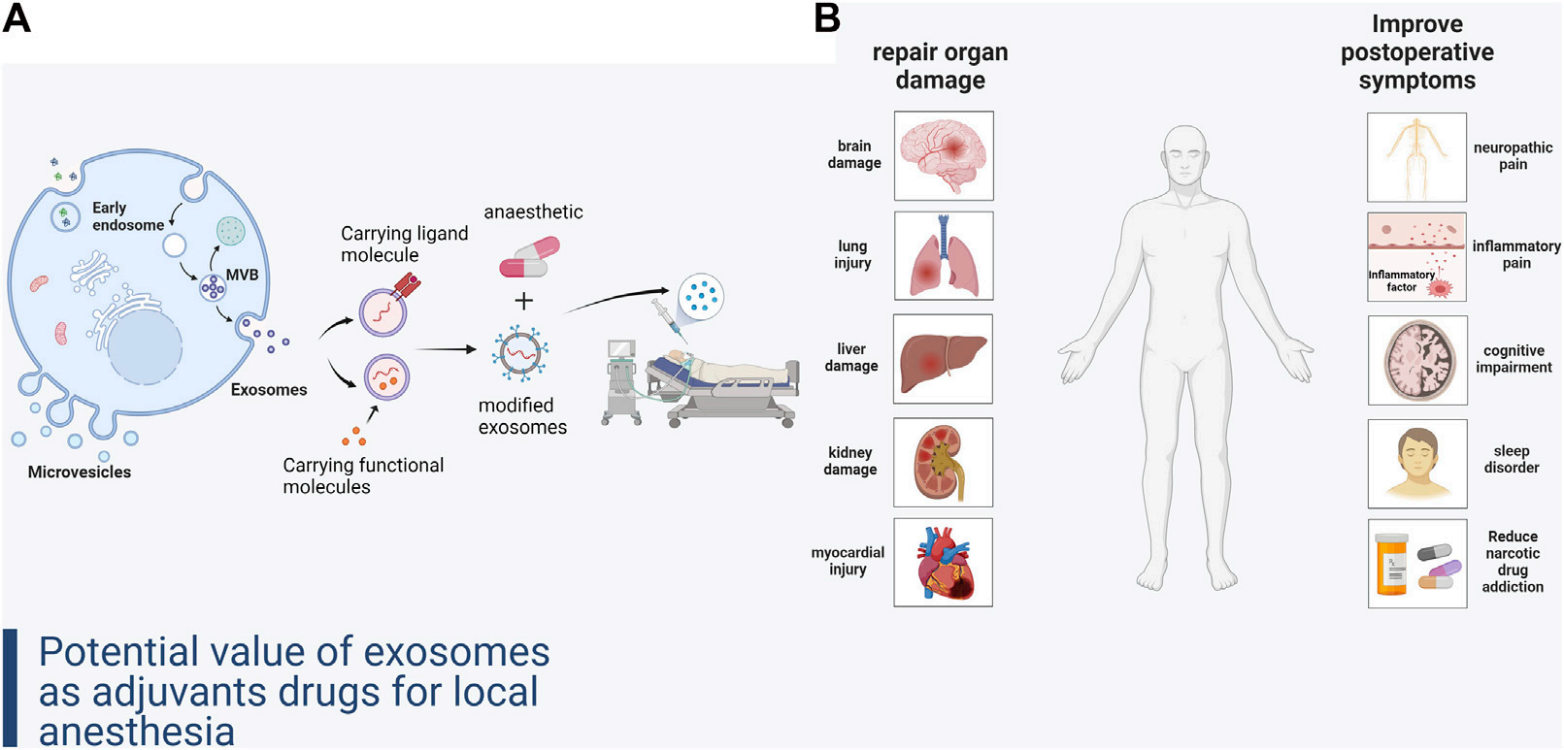
**(A)** Formation of exosomes and construction of local anesthetic adjuvant-like exosomal drugs. **(B)** Improvement of organ damage and complications after local anesthesia by exosomes.

The common side effects of local anesthetic adjuvants limit their clinical use. These side effects may pose an even greater threat in certain procedures involving organ damage, such as cardiopulmonary surgery which requires the establishment of extracorporeal circulation (Edinoff et al., 2021). Prevention and repair of organ damage due to some common surgical procedures is also a limitation of current anesthetic-assisting drugs. Therefore, development of novel anesthetic-assisting drugs is a key research imperative in the field of anesthesia today. Research on the drug delivery and signaling function of exosomes in parenchymal organ protection and remodeling, accelerating postoperative recovery, and reducing complications may provide new insights for the development of novel local anesthetic adjuvant drugs (Huang et al., 2017) (Figure 1).

### 3.1 Protection and remodeling of multiple organs

Ischemia-reperfusion (I/R) injury is a key consideration in a variety of surgical procedures, and both temporary blockage of blood flow and restoration of blood supply can have detrimental effects on cellular function and structure. In the early ischemic phase of the organ, this will lead to hypoxia and malnutrition, while after prolonged ischemia, the cellular metabolites can cause metabolic acidosis. After the restoration of the blood supply, local inflammation and increased reactive oxygen species (ROS) lead to secondary cellular damage (Wu et al., 2018). This may be associated with cellular autophagy, apoptosis, necrosis, and necroptosis (Lopez-Neblina et al., 2005; Gottlieb, 2011; Zhu et al., 2016). Although short-term ischemia causes limited damage to organs, it is not clear exactly when I/R injury is classified in the long and short term in individual organs and how difficult it is to restore organ blood supply promptly when dealing with complex procedures. Therefore, the most severe possible scenarios, such as ischemic necrosis of organs, should be taken into account during the prevention and treatment phases of I/R injury. Drugs targeting cell regeneration and repair may help address this challenge. Exosomes derived from different tissue cell sources in specific states have been shown to exhibit intervention and repair functions after I/R injury in a variety of organs.

#### 3.1.1 Myocardial injury

Organs stimulate their own protective response in the presence of ischemia. Ischemic preadaptation may represent this phenomenon, wherein transient ischemia of the heart renders the heart resistant to subsequent lasting ischemic injury (Huang et al., 2021; Wei et al., 2021). In animal models of I/R studies, stem cell injection was shown to induce the formation of new cardiomyocytes. However, the vast majority of these new cardiomyocytes were not differentiated from the injected stem cells, but rather from the endogenous stem cells of the heart. Further studies showed that *ex vivo* stem cell exosomes stimulate the formation and differentiation of endogenous stem cells. This suggests that the protective mechanisms of the ischemic organ originate from the organ itself and can be activated by stem cell exosomes (Wei et al., 2021). In addition, exosomes released from immune cells are also involved in the process of resistance to ischemic injury, such as the release of HP70-containing exosomes from dendritic cells that stimulate the PI3K/mTOR axis to regulate Treg and Th17 cell differentiation and attenuate I/R injury (Lai et al., 2010). Cardioprotective miRNAs in exosomes released from cardiomyocytes subjected to transient ischemia showed the ability to downregulate protein expression by binding to mRNAs in the cytoplasm or upregulate protein production by impairing the expression of repressive genes, thereby attenuating the damage after cardiac ischemia (Lu and Rothenberg, 2018). During episodes of ischemic myocardial injury, cardiomyocyte-derived exosomes enriched in miR-133a and miR-1 show potent cardioprotective functions by limiting cardiomyocyte hypertrophy (Pan et al., 2012; Soufi-Zomorrod et al., 2016). Since autophagy and apoptosis are important pathogenic mechanisms in ischemic cardiomyopathy, interfering with autophagic and apoptotic processes may be an important direction to ameliorate post-ischemic cardiac injury. During the acute phase of cardiac ischemia, cardiomyocytes were found to release cardioprotective exosomes enriched in the regulation of autophagy and apoptosis, such as miR-214, an exosome that prevents apoptosis and loss of contractility, and miR-30a, an exosome that regulates autophagy (Sun et al., 2018; Zhang et al., 2019). Furthermore, when plasma exosomes derived from habitual exercisers were injected into rats with myocardial infarction, the area of myocardial infarction was significantly reduced compared to that in rats injected with exosomes from non-exercising subjects. This suggests a potent cardioprotective effect of exosomes derived from exercising humans (Hou et al., 2019). Exosomes from cardiac tissue also play an important role in maintaining cardiovascular homeostasis. In other cardiac disease processes that affect the homeostasis of the cardiovascular system (e.g., arrhythmias, cardiac hypertrophy, and cardiomyopathy), exosomes released from specific cells may improve cardiomyocyte dysfunction due to certain miRNA dysfunctions (Wei et al., 2021).

#### 3.1.2 Kidney injury

Renal injury is also a relatively common complication of surgical procedures. Studies have demonstrated the value of MSC exosomes in improving the treatment of acute kidney injury (AKI) induced by I/R, nephrectomy, and drug toxicity (gentamicin) (Bruno et al., 2012; He et al., 2012; Zou et al., 2014). *In vivo* experiments, MSC exosomes were found to improve AKI recovery by preventing apoptosis and increasing renal tubular epithelial cell proliferation (Bruno et al., 2009). During tissue repair, exosomes promote the expression of anti-apoptotic genes (*Bcl2*, *Bcl-XL*, and *BIRC8*) in renal tubular epithelial cells and downregulate the expression of pro-apoptotic genes (*Casp8, Casp1*, and *LTA*) (Bruno et al., 2012). MSC exosomes can also induce phosphorylation and subsequent activation of extracellular regulated kinase (ERK)½ to stimulate renal cell proliferation (Zhou et al., 2013). Reversal of renal function and morphology after multiple injections of MSC exosomes ultimately eliminates renal fibrosis and reduces mortality. Exosomes showed potent renoprotective effects due to transporting insulin-like growth factor-1 receptor (IGF-1R) mRNA to renal tubular cells (Tomasoni et al., 2013). The acute I/R phase in any tissue/organ always triggers a rapid and intense inflammatory response, including the recruitment of inflammatory cells and the production of cytokines, free radicals, and oxidative stress (Chen et al., 2014; Zhao et al., 2016). These cellular-molecular perturbations in turn are directly involved in erythrocyte injury and further exacerbating tissue/organ damage (Chen et al., 2013). Therefore, inhibition of inflammatory response and oxidative stress is crucial to ameliorate acute IR injury. An increasing body of evidence shows that MSC-derived exosomes have immunomodulatory and paracrine effects, improve organ function after injury in preclinical studies, and that adipose-derived MSC-derived exosomes and stem cell therapy are equally effective in reducing inflammation, oxidative stress, and deterioration of renal function (Dorronsoro and Robbins, 2013; Fleig and Humphreys, 2014; Zou et al., 2014; Chang et al., 2015; Ko et al., 2015).

#### 3.1.3 Lung injury

The lung is also a common site of damage after anesthesia and surgical procedures. MSC exosomes also show a protective function in pulmonary I/R. In an *in vitro* human pulmonary I/R model, MSC exosomes showed a dose-dependent increase in the clearance of fluid from the alveoli leading to a reduction in lung weight after perfusion and ventilation, and ultimately improved airway and hemodynamic parameters (Lee et al., 2013; Gennai et al., 2015). Interestingly, co-administration of exosomes with anti-CD44 antibodies attenuated these effects, suggesting that CD44 receptors on the surface of exosomes play a key role in the entry of exosomes into injured lung cells and their effects (Gennai et al., 2015). Moreover, during the initial phase of lung injury, MSC exosomes exhibit interference with hypoxic signaling, inhibit hypoxia-induced mitogenic factor expression, and activate alveolar macrophages (Lee et al., 2012). However, conditioned media depleted of exosomes did not have a similar effect, suggesting that exosomes may play a role in ameliorating lung injury rather than the soluble molecules released by stem cells (Lee et al., 2012). Furthermore, in a mouse model of *E. coli* pneumonia, administration of MSC exosomes was shown to significantly improve survival, suggesting their potential value in the treatment of pneumonia. This may be related to the fact that exosomes alleviate lung inflammation, restore lung protein permeability, and inhibit bacterial growth (Monsel et al., 2015). Furthermore, MSC-derived exosomes had an equivalent effect in ameliorating acute lung injury due to severe bacterial pneumonia compared to their parental stem cells (Lee et al., 2009; Zhu et al., 2014). Since exosomes exhibit immunosuppressive effects similar to those of stem cells, researchers further verified whether exosomes have similar repair functions in allergic pneumonia. In the established preclinical model of allergic airway inflammation caused by mucosal sensitization and mycelial Aspergillus extract stimulation mesenchymal stem cell exosomes did exhibit a reparative effect (Cruz et al., 2015). This effect was likely related to the attenuation of Th2/Th17-mediated airway hyperresponsiveness leading to reduced pulmonary inflammatory response (Cruz et al., 2015). Stem cell exosomes have shown more significant effects than the existing drugs used for the prevention and treatment of pulmonary fibrosis. In a model of silica-induced lung inflammation and fibrosis, Phinney et al. (2015) found that MSC exosomes could regulate toll-like receptor signaling and cytokine secretion in macrophages through the transfer of regulatory microRNAs. The presence of miR-451, which is abundant in MSC exosomes and inhibits TNF and macrophage migration inhibitory factors, suggests that miR-451 is transferred to macrophages by exosomes and thus functions to reduce lung fibrosis (Liu et al., 2022). Also, MSC-derived exosomes prevented monocyte recruitment, which reduced the secretion of IL-10 and TGFβ associated with lung fibrosis.

#### 3.1.4 Other organs

Interestingly, one study found significant expression of enzymes involved in glycolysis in MSC exosomes by mass spectrometry and antibody arrays (Lai et al., 2013). These enzymes contained in MSC exosomes inhibited epithelial-mesenchymal transition and collagen production in hepatocytes to ameliorate liver fibrosis. Tan et al. (2014) observed the protective effects of MSC exosomes against acetaminophen- and hydrogen peroxide-induced liver injury, which were mainly due to an increase in hepatocyte proliferation, as evidenced by elevated levels of proliferating cell nuclear antigen, and increased cell viability. In a rat model of neural I/R, exosomes were found to transfer miR-133b from MSCs to injured neuronal cells, ultimately promoting post-stroke neuronal remodeling and functional recovery (Xin et al., 2012; Xin et al., 2013a). In rats with middle cerebral artery occlusion, administration of therapeutic amounts of mesenchymal stem cell exosomes led to significant acceleration in the recovery of brain function and the rate of neurosynaptic remodeling, neurogenesis, and angiogenesis. This may be related to the fact that exosomes increase the number of cells releasing bicortin (a marker of neuroblasts) and vascular hemophilia factor (a marker of endothelial cells) (Xin et al., 2012; Xin et al., 2013a). In conclusion, MSC exosomes may hasten the postoperative recovery from traumatic brain injury (cerebral hemorrhage, cerebral hematoma); in addition, the ability of exosomes to function directly across the blood-brain barrier avoids the potential risks resulting from overdosing (Morad et al., 2019). The available evidence suggests that use of exosomes for promoting neurorepair is worthy of further research.

In summary, exosomes have similar physiological pathways in the prevention and repair of multiple organ injuries, which will facilitate their use as local anesthetic adjuvants to improve multiple postoperative organ injuries. It also avoids the duplication of exosome drug development, waste of materials, and the burden of use for anesthesiologists. The use of exosomes for a variety of functions such as cellular remodeling, inflammation inhibition, reduction of hypoxic injury, and relief of fibrosis has made it possible to develop an exosomal biologic agent for the prevention and repair of multiple organ injuries (Kalluri and LeBleu, 2020). Exosomal anesthetic adjuvants may be more promising than the existing local anesthetic adjuvants due to the organ repair functions of exosomes derived from specific cell subsets (currently, mesenchymal stem cells are mainly considered).

### 3.2 Improve postoperative adverse effects to improve anesthetic efficacy

Improving postoperative complications is the primary purpose of use of anesthetic adjuvants. Therefore, the selection of new local anesthetic adjuvants should be based on addressing the effects of current anesthetic drugs as well as the postoperative complications resulting from surgical procedures in order to accelerate postoperative recovery. It has been found that exosomes released from specific tissue cells not only remodel parenchymal organ damage, but also promote the efficacy of anesthetic drugs as well as mitigate their side effects (Xin et al., 2013b; Yubo et al., 2017). This may be related to the inhibitory effect of exosomes on the release of inflammatory factors as well as the nerve impulses. In addition, exosomes may also play a key role in postoperative chronic pain as well as in the process of nerve injury (Xin et al., 2013a). Therefore, some common adverse events associated with anesthesia (e.g., cognitive impairment and drug addiction) can be avoided by systemic or local administration of exosomes of specific cellular origin or their artificial modifications.

#### 3.2.1 Reduces post-operative pain

##### 3.2.1.1 Inflammatory pain

It is well known that inflammatory processes are involved in the pain response of the body and a large number of cytokines, chemokines, and other factors are involved in the development of inflammatory pain (Seifert and Baerwald, 2021). Local or systemic discomfort in post-surgical patients is mostly associated with the release of local inflammatory factors in tissues, such as PGE2, bradykinin, and substance P. Other inflammatory factors associated with tissue damage such as nitric oxide and lysosomal enzymes can also trigger a more powerful inflammatory response further exacerbating the release of cytokines associated with pain (Zhang and An, 2007). This may be related to the fact that the inflammatory response can cause persistent sensitization of peripheral and central nerves to painful stimuli (Harth and Nielson, 2019). Therefore, reducing local inflammatory cytokine accumulation is one of the important ways to reduce postoperative pain. There is growing evidence that exosomes may contribute to the release of factors from local inflammation-associated cells in tissues to reduce the inflammatory response and thus relieve pain. Exosomes have been shown to inhibit the number of sensitizing endings released by cells and reduce the release of neuroinflammatory sensory mediators (Gupta and Pulliam, 2014). Exosomes from bone and adipose tissue mesenchymal cells were also found to significantly reduce the release of IL-1β, IL-6, and PGE2 while promoting IL-10 formation in studies (Tofiño-Vian et al., 2017). In the same study, analysis of serum-derived exosomes from patients with chronic pain syndrome (CRPS) revealed that 127 miRNAs were significantly differentially expressed compared to control-derived exosomes. Among them, three miRNAs (miR-21-3p, miR-146a and miR-146b) known to be involved in the control of overactivation of innate immune response were overexpressed in both mouse and human models (McDonald et al., 2014). Uncontrolled or unresolved inflammation may be an active pathway of systemic inflammation involved in the pathogenesis of several pain disorders, such as osteoarthritis, rheumatoid arthritis (RA), inflammatory bowel disease (IBD), and neurodegenerative diseases (McDonald et al., 2014).

Indeed, exosomes derived from mesenchymal stem cells are responsible for the improvement of pain after intra-articular injections in patients with osteoarthritis (Yubo et al., 2017). This is related to the reduction of pro-nociceptive cytokines, such as IL-1β, by exosomes from bone marrow stem cells. Furthermore, in osteoarthritis, exosomes derived from bone marrow mesenchymal stem cells (BMSCs) have been found to relieve pain. Exosomes from human embryonic stem cell-induced mesenchymal stem cells (ESC-MSCs) may alleviate osteoarthritis and thus joint pain by balancing the synthesis and degradation of the extracellular matrix of cartilage (Wang et al., 2017a). In models of colitis, miR-219-containing lactic endogenous exosomes reduce inflammation by inhibiting the TLR-4/NF-kB signaling pathway (Xie et al., 2019). Notably, exosomes derived from mesenchymal stem cells also reversed chronic inflammatory pain in animals treated with complete Freund’s adjuvant (Pan et al., 2014). Moreover, exosomes can also contain many antinociceptive molecules that can slow down nociception when delivered to the target cells. For example, circulating exosomes expressing Annexin A-1 (Ayoub et al., 2008), which is antinociceptive and has potent anti-inflammatory effects at the spinal cord level, are increased in patients with inflammatory bowel disease (Leoni et al., 2015), and exosomes expressing miR-216a-5p have also been shown to improve neuropathic pain in animals with sciatic nerve compression (Wang et al., 2017b). Interestingly, antinociceptive molecules have also loaded into exosomes and injected into nerve sheaths to improve neuropathic pain. For example, exosomes from human umbilical cord stem cells have shown a role in preventing or reducing sensory deficits in rats with spinal nerve ligation. Intrathecal administration of exosomes was reported to improve neuropathic pain behavior by acting on dorsal root ganglion neurons and glial cells of the ganglion and spinal cord (Shiue et al., 2019). In conclusion, exosomes are potentially valuable either as direct targeting agents or drug delivery vehicles, in inhibiting the release of inflammatory factors, delivering antipain molecules, or delivering analgesic drugs. It can be hypothesized that exosomes may also be able to alleviate somatic pain in post-anesthesia patients by improving the inflammatory response.

##### 3.2.1.2 Neuropathic pain

In terms of neuropathic pain, proteomic characterization of exosomes from a mouse model of neurological injury (SNI) suggests that cargo sorting of vesicular proteins is a critical step in mediating the underlying signaling mechanisms of neuropathic pain. Significant upregulation of complement component 5a (C5a) and intercellular adhesion molecule 1 was detected in exosomes from the SNI model compared to controls. Ex-SC attenuated SNL-induced pain and enhanced axonal regeneration of injured nerves in rats. MSC exosomes contain proteins and functional RNAs that exert neuroprotective and regenerative effect on damaged tissues. In addition, the analgesic effect of Ex-SC remained evident at day 21 after ligation, suggesting that Ex-SC can induce long-lasting analgesic effects (Kusuhara et al., 2019). Many studies on CRPS have also highlighted the involvement of exosomes in neuropathic pain. For example, a single injection of macrophage-derived exosomes was found to alleviate thermal nociceptive hyperalgesia (Jean-Toussaint et al., 2020). Indeed, neurons can also release exosomes from dendrites and soma and are regulated by glutamate and intracellular Ca2+. It is hypothesized that exosome release during neuronal depolarization could be actively involved in synaptic plasticity (Goldie et al., 2014). Neurons are also receptor cells for exosomes released from a variety of non-neuronal cells. Upon exposure to exosomes, neurons can undergo changes in synaptic strength and number as well as in cell survival and metabolism (Lopez-Verrilli et al., 2013; Simeoli et al., 2017; Amit et al., 2020; Li et al., 2020).

Interestingly, neurons themselves can also exchange synaptic information *via* extracellular vesicles and neuronal exosome content can regulate the number of synapses between neurons (Men et al., 2019). For example, exosome PRR7 from hippocampal neurons can be taken up by neighboring neurons, reducing the number of excitatory synapses (Lee et al., 2018). This suggests the involvement of exosomes in the whole process of neuronal cell activity. We can hypothesize that exosomes can trigger functional and phenotypic changes in sensory neurons because of the presence of a large number of nociceptive molecules in their cargo. Indeed, intrathecal injection of exosomes from hypoxia-pretreated neurons did reduce neuropathic pain in rats with spinal cord injury (Wang et al., 2021a). The protective effect may be mediated by miR-126-3p in exosomes, which is associated with neuropathic pain. Therefore, restoring miR-126-3p levels after spinal cord injury or peripheral nerve injury through exosomal delivery may be a potential therapy for post-injury neuropathic pain.

##### 3.2.1.3 Chronic pain

Postoperative patients with chronic pain typically experience depression, anxiety, sleep disturbances, and abnormal decision-making; these conditions exacerbate the severity of pain and reduce the quality of life (Davis et al., 2016; Gerdle et al., 2019). Chronic neuropathic pain has been shown to severely distress the mental state of postoperative patients and affects the efficiency of surgical recovery (Knopp et al., 2019). Neuropathic pain is a chronic pain disorder that can lead to sensory, motor, and autonomic dysfunction as well as abnormal pain, nociceptive hyperalgesia, dystonia, and tremor (Finnerup et al., 2021). NSAIDs and opioids are the main classes of drugs used for chronic neuropathic pain; however, they have limited efficacy and cause serious side effects.

Indeed, studies on different chronic pain disorders have demonstrated the involvement of exosomes in the pain process and they are considered as a promising therapeutic approach to improve pain with fewer side effects. Exosomes from mesenchymal stem cells (ex-MSCs) have been shown to alleviate pain symptoms with fewer side effects in several chronic pain models (Ren et al., 2019). Attempts have been made to use MSC-derived exosomes as a treatment for peripheral nerve injury. Exosomes can transfer their miRNA cargo to target neurons promoting axonal growth and nerve survival (Dong et al., 2019). However, the specific mechanism of action of these exosomes is not known and may be related to the presence of many neurotrophic factors among the cargo of MSC exosomes, such as fibroblast growth factor-1 (FGF-1), glial cell-derived neurotrophic factor (GDNF), insulin-like growth factor-1 (IFG-1), brain-derived neurotrophic factor (BDNF), and nerve growth factor (NGF). These factors help maintain the establishment of the repair microenvironment of peripheral nerves after injury (Kosik, 2006). This is also due to the ability of exosomes from regenerative stem cells or neuronal cells to cross the blood-brain barrier and have neuroprotective and tissue repair effects (Xin et al., 2013b). This ability suggests the possibility of using exosomes to transfer specific drug molecules acting on target tissues to address brain injury.

#### 3.2.2 Relief of post-operative psychiatric disorders

##### 3.2.2.1 Postoperative cognitive impairment

A potential association between use of general anesthesia for surgery in elderly and postoperative cognitive impairment was first reported in 1955 (Bedford, 1955). Since then, attention has focused on the cognitive effects of general anesthesia, including delirium, postoperative cognitive dysfunction, the development of dementia, and cognitive decline in preoperative pre-existing dementia. Postoperative cognitive dysfunction is often defined as measurable cognitive impairment over time, as assessed by neuropsychological tests, which may affect memory, attention, and psychomotor function (Lin et al., 2020). In the case of surgical procedures, this will also severely impact the patient’s postoperative recovery (Rundshagen, 2014). With an aging population and the increasing prevalence of Alzheimer’s disease and other forms of dementia, cognitive impairment in the elderly after general anesthesia is likely to occur more frequently in the future in clinical practice (Belrose and Noppens, 2019). There is a need to understand the underlying mechanisms of perioperative cognitive impairment. Currently, it is believed that the underlying pathology of neurologically related disorders may increase the susceptibility to the potential neurotoxic effects of surgical stress and/or anesthesia exposure and increase the risk of cognitive impairment progression (Needham et al., 2017). This may involve multiple mechanisms, including altered Aβ processing or accumulation, pathological changes in tau, neuroinflammation, calcium dysregulation, and mitochondrial dysfunction, with altered synaptic structure or function, calcium dysregulation, and mitochondrial dysfunction being more clearly associated with the development of cognitive impairment following anesthesia and/or surgery (Belrose and Noppens, 2019).

Interestingly, increased expression of P-gp receptors after use of recombinant brain microvascular endothelial cell exosomes revealed that exosomes could enter cells *via* endocytosis and prevent lysosome-mediated degradation of P-gp receptors (Pan et al., 2020). In mice, increased levels of intracellular P-gp receptors decreased Aβ in the hippocampus and ameliorated cognitive deficits caused by Aβ aggregation. Compared to exosomes from children with normal cognitive function, plasma exosomes from children in the experimental group showed altered morphology of ZO-1, increased permeability of the blood-brain barrier, which indirectly affected the microenvironment and neural networks of the brain (Pan et al., 2020). In an APP/PS1 mouse model of Alzheimer’s disease, repeated intracerebral treatment with MSC-exosomes early in the disease also resulted in a reduction of Aβ plaques in the brain (Ding et al., 2018), and exosomes isolated from N2a neuroblastoma cell cultures or human cerebrospinal fluid were shown to reduce synaptic plasticity defects and restore normal long-term enhancement (An et al., 2013). In addition, exosomes have shown more pronounced repair functions in neurological disorders such as stroke, traumatic brain injury, and certain types of epilepsy (Kalluri and LeBleu, 2020). Therefore, cell-specific exosomes, especially MSC-exosomes, may be a potential alternative to dexmedetomidine to reduce the occurrence of postoperative cognitive impairment. Furthermore, exosomes may be more beneficial for regenerative repair of pre-existing neurological tissue damage compared to dexmedetomidine. In addition, exosomes act as naturally occurring biologic agents that freely penetrate the blood-brain barrier and can be modified to be loaded with therapeutic molecules, thus acting as efficient carriers of therapeutic agents to the brain. There is great promise in the use of exosomes as drugs to improve postoperative cognitive impairment.

##### 3.2.2.2 Post-operative sleep disorder

Another anesthesia-related psychiatric disorder that plagues postoperative recovery is postoperative sleep disturbance, which often occurs in elderly patients after major surgery and has a negative impact on postoperative recovery. Postoperative sleep disorders can increase the incidence of postoperative fatigue, severe anxiety and depression, pain sensitivity, and cognitive dysfunction, which can cause or exacerbate neurodegenerative disease through amyloid aggregation and tau protein accumulation (Holth et al., 2019). The occurrence of sleep disorders is associated with a variety of factors, including age, preoperative complications, type of anesthesia, degree of surgical trauma, and postoperative pain (Shokri-Kojori et al., 2018; Bah et al., 2019). Postoperative sleep disorders severely affect the quality of life and increase the difficulty and burden of care; thus, medications suggested to improve the sleep environment to alleviate sleep disorders in postoperative patients have been used to improve sleep after discharge, including zolpidem, melatonin, and/or dexmedetomidine (Su and Wang, 2018). This was shown to reduce the incidence of mental disorders and cardiovascular events, improve prognosis, and shorten the length of hospital stay (Su and Wang, 2018). In addition, exosomes may also slow down end-organ lesions caused by sleep disorders. For example, exosomes contribute to the delivery of therapeutic genetic material, such as microRNAs and proteins, to exert neuroprotective effects and reduce cognitive impairment (Cui et al., 2019; Chen et al., 2020). Interestingly, plasma exosomes obtained from mice with sleep rhythm disorders showed significantly reduced expressions of Bmal1, Cry2, and Per1 (Khalyfa et al., 2017). In addition, tau proteins associated with neurological damage due to postoperative sleep disorders can also be transmitted between neurons *via* exosomes (Wang et al., 2017c). It is suggested that exosomes can act as a bridge between peripheral clock control genes and central rhythms, transmitting the effects of circadian rhythm disorders to target organs, while participating in the cognitive decline caused by sleep disorders. Therefore, interfering with the production of externally relevant exosomes may be one of the ways to effectively suppress the frequency of postoperative sleep disorders.

#### 3.2.3 Suppression of postoperative inflammatory syndrome

Exosomes also inhibit the development of severe postoperative inflammatory syndrome, an excessive inflammatory response involving the innate immune system, neutrophil and macrophage accumulation, cytokine secretion, T and B cell recruitment and antibody formation in an attempt to eliminate pathogenic pathogens. However, this process also leads to bystander attack on major organs/tissues, resulting in ineffective host defense mechanisms, rapid organ failure, and potential death (Chang et al., 2018; Feng et al., 2022). Fortunately, much evidence suggests that stem cells, especially adipose-derived mesenchymal stem cells (ADMSCs), have an intrinsic immunomodulatory capacity (Le Blanc et al., 2003; Sun et al., 2011). In a study, septic animals treated with healthy ADMSC-derived or apoptotic ADMSC-derived exosomes showed significantly lower mortality rate compared to their untreated counterparts; furthermore, exosome treatment was found to protect the lungs and kidneys against sepsis syndrome-induced injury (Chang et al., 2018). This suggests that exosomes can effectively suppress both the excessive inflammatory response after anesthesia as well as ameliorate organ tissue damage resulting from immune storms, especially with respect to malignant hyperthermia and allergic reactions caused by anesthetic drugs.

#### 3.2.4 Increased drug tolerance

In addition, exosomes have been shown to modulate the tolerance of anesthesia-related analgesic drugs. For certain large, traumatic surfaces in postoperative recovery, larger doses are required to achieve the desired analgesic effect, and large doses of morphine can exacerbate adverse drug reactions (Mazoit et al., 2007). Morphine tolerance is defined as a decrease in the effectiveness of opioids in controlling pain. This means that to achieve the same analgesic effect, the dose must be increased over time (Kliewer et al., 2020). In a study, mRNA levels of spinal exosomal protein-related genes were found to be significantly elevated in morphine-tolerant rats, and administration of exosomal protein inhibitors prolonged morphine analgesia and alleviated morphine tolerance and behavioral nociceptive hyperalgesia in rats. Therefore, a morphine analgesic tolerance model based on the exosomal protein inhibitor 4-PBA or TUDCA was proposed, and intrathecal administration of the exosomal protein inhibitor 4-PBA or TUDCA was found to enhance morphine analgesia. The glutamate transporter protein GLT-1 is known to play a crucial role in glutamatergic regulation of glutamate physiological homeostasis, neurotoxicity, and opioid tolerance. In contrast, intrathecal injection of the exosomal protein inhibitors TUDCA or 4-PBA at the protein level prevents morphine-induced downregulation of the glutamate transporter GLT-1, which mediates the body’s tolerance to opioids (Lyu and Li, 2022).

## 4 Prospects

The key challenges in the development of local anesthetic adjuvant drugs is the duplication of action, high incidence of side effects, and inadequate efficacy. Because of their inadequate efficacy, much of the contemporary research on anesthetic drugs has focused on the development of anesthetic drugs themselves with fewer side effects. This has led to a vicious cycle of research on local anesthetic adjuvants. However, it is undeniable that, as stated above, local anesthetic adjuvants are still necessary to hasten the onset of action of anesthetic drugs and reduce side effects in the absence of fundamental changes in anesthetic drugs. Therefore, the development of more affine, efficient, and less toxic anesthetic adjuvants remains an important issue in the development of anesthetic drugs. The somatic affinity, cellular repair ability, and similar and more efficient amelioration of post-anesthetic complications exhibited by exosomes suggests their potential as a new direction for anesthetic adjuvant development. However, there are still some issues that need to be addressed before developing exosomal products for anesthetic adjuvants or translating exosomes into clinical applications.

An important issue is that current research on exosomes as a way to alleviate postoperative complications of anesthesia involves multiple specific cell sources. For example, improvement of cardiac function was derived from exosomes released from cardiomyocytes after exercise (Hou et al., 2019), repair of lung injury was mainly derived from exosomes released from mesenchymal stem cells (Gennai et al., 2015), and reduction of postoperative pain was associated with exosomes of macrophage origin (Jean-Toussaint et al., 2020). The use of diverse materials enhances the complexity and costs of the development of new anesthetic adjuvants. Of course, there is an increasing focus on the development of MSC exosomal drugs due to the advantages of a wide source of MSC exosomes, their diverse efficacy, and definitive studies (Xunian and Kalluri, 2020). Furthermore, studies on exosomes as drug carriers have made it possible to load molecules with specific effects to complement the function of exosomes. For example, loading miRNA cargoes for transfer to target neurons can promote the repair of damaged neurons. Loading deletion proteins or primitive proteins prior to pathological alterations can be used to improve symptoms and stop disease progression in Alzheimer’s disease and other forms of dementia (Wang et al., 2021a). Therefore, MSC-derived exosomal drugs loaded with multiple therapeutic molecules may be the primary direction for exosomal drug development. At the same time, the paucity of studies elucidating the specific biological effects of exosomes of relevant origin makes their safety and reliability controversial (Choi et al., 2022). This requires a more detailed elucidation of the role of the biomolecules contained in exosomes of specific cellular origin in order to avoid their potential biohazards.

Another challenge is the short preservation half-life of primary cell-derived exosomes and their insufficient retention in target tissues *in vivo*. The integrity of cell-derived exosomes has been found to decrease exponentially with time. For example, freshly extracted exosomes lasted at most 30 min at room temperature and only 4–5 h in a 4-degree refrigerator; there was a more pronounced decrease in the number of intact exosomes after melting at −80° and using specific solutions such as PBS solution containing fetal bovine protein, although they lasted 6–8 months (Zhang et al., 2020). Therefore, development of methods for more durable preservation of exosomes is a key research imperative. In addition, different parental cell types and drug delivery methods can cause significant differences in the degradation rate of exosomes *in vivo*, which increases the dosage of exosomal drugs and the risk of potential complications (Wang et al., 2021b). In this regard, in order to improve the retention of exosomal drugs in the target organ, delivery methods that are closer to the target organ can be chosen, such as transnasal sprays or endoscopic injections for the treatment of lung injury. Exosomes can also be modified by modifying their surface proteins or carrying ligand proteins to make the exosomes more concentrated to increase the target organ absorption rate. At the same time, exosomes modified with targeted ligands can be used at an early stage of organ damage induced by anesthetic drugs and alleviate the clinical symptoms. This would also help meet the anesthesiologist’s requirement for fast-acting anesthetic adjuvants.

Finally, most of the studies on exosomes are based on animals (mainly rats and mice). Although a small number of studies on MSC exosomes involved human-derived *in vitro* studies, these models may not fully mimic the pathophysiological processes of human patients due to the vast differences in genetic information between humans and animals. Therefore, whether these exosomally contained molecules exist within human-derived cellular exosomes or whether similar functions exist remains to be further explored. Furthermore, owing to the diversity of disease mechanisms, such as the involvement of multiple cellular and molecular pathways, a single therapeutic target may not ameliorate the clinical symptoms or reverse the disease progression. Therefore, specifying the major biomolecules involved in the disease may make it easier to engineer exosomes. This can also be addressed by engineering exosomes to carry multiple biomolecules.

## 5 Conclusion

In conclusion, exosomes may be good targets for future local anesthetic adjuvant drug development based on their role in ameliorating adverse reactions and repairing organ damage caused by anesthetic drugs. The good bioaffinity of exosomes as well as their specific targeting ability and modifiability make them promising candidates for further research. Clinical application of exosomes requires breakthroughs in exosome extraction, preservation, and bioengineering technologies. Elucidation of the detailed mechanisms of anesthetic adverse reactions and the specific principles of exosome resistance to their adverse reactions is required to enable large-scale production of highly efficient engineered local anesthetic adjuvant-like exosomal drugs.
